# Assembly and Analysis of Differential Transcriptome Responses of *Hevea brasiliensis* on Interaction with *Microcyclus ulei*

**DOI:** 10.1371/journal.pone.0134837

**Published:** 2015-08-19

**Authors:** Uriel Alonso Hurtado Páez, Ibonne Aydee García Romero, Silvia Restrepo Restrepo, Fabio Ancizar Aristizábal Gutiérrez, Dolly Montoya Castaño

**Affiliations:** 1 Laboratorio de Caracterización Molecular, Instituto de Biotecnología, Universidad Nacional de Colombia, Bogotá, Colombia; 2 Laboratorio de Micología y Fitopatología, Universidad de los Andes, Bogotá, Colombia; 3 Grupo de Bioprocesos y Bioprospección, Instituto de Biotecnología, Universidad Nacional de Colombia, Bogotá, Colombia; RIKEN Biomass Engineering Program, JAPAN

## Abstract

Natural rubber (*Hevea brasiliensis*) is a tropical tree used commercially for the production of latex, from which 40,000 products are generated. The fungus *Microcyclus ulei* infects this tree, causing South American leaf blight (SALB) disease. This disease causes developmental delays and significant crop losses, thereby decreasing the production of latex. Currently several groups are working on obtaining clones of rubber tree with durable resistance to SALB through the use of extensive molecular biology techniques. In this study, we used a secondary clone that was resistant to *M*. *ulei* isolate GCL012. This clone, FX 3864 was obtained by crossing between clones PB 86 and B 38 (*H*. *brasiliensis* x *H*. *brasiliensis*). RNA-Seq high-throughput sequencing technology was used to analyze the differential expression of the FX 3864 clone transcriptome at 0 and 48 h post infection (hpi) with the *M*. *ulei* isolate GCL012. A total of 158,134,220 reads were assembled using the *de novo* assembly strategy to generate 90,775 contigs with an N50 of 1672. Using a reference-based assembly, 76,278 contigs were generated with an N50 of 1324. We identified 86 differentially expressed genes associated with the defense response of FX 3864 to GCL012. Seven putative genes members of the AP2/ERF ethylene (ET)-dependent superfamily were found to be down-regulated. An increase in salicylic acid (SA) was associated with the up-regulation of three genes involved in cell wall synthesis and remodeling, as well as in the down-regulation of the putative gene *CPR5*. The defense response of FX 3864 against the GCL012 isolate was associated with the antagonistic SA, ET and jasmonic acid (JA) pathways. These responses are characteristic of plant resistance to biotrophic pathogens.

## Introduction

The production of natural rubber (*Hevea brasiliensis*) in Latin America has increased in the last decade. Countries such as Brazil and Guatemala are the main producers, yielding 178,500 tons/year and 93,600 tons/year, respectively [1]. Colombia has also increased its planting area by 86.4%. In 2002, Colombia had 6,000 ha of available planting area and now counts 44,100 ha, of which 4,000 ha are currently being used for production [2]. This increase is due in large part to the support of the state, which seeks to replace illegal crops and position the country as the third largest producer of raw materials in Latin America [3]. However, the continent accounts for only 3% of the global natural rubber production compared with Southeast Asian countries such as Thailand, Indonesia and Malaysia, which together produce 92% of the natural rubber in the world [1]. This difference is mainly due to South American leaf rubber blight (SALB) caused by the ascomycete fungus *M*. *ulei*, which only infects some species of the genus *Hevea*. This disease is endemic in countries that cultivate rubber in Latin America and has restricted cultivation to areas known as “climate escape areas”. These areas are characterized by long periods of drought and low relative humidity during the refoliation period of the tree, which limits the development of *M*. *ulei* [4].

Following the devastation caused by *M*. *ulei* in natural rubber plantations established in the state of Para, Brazil, in the first half of the 20^th^ century, the presence of some disease-resistant *Hevea* species were observed. These clones were used to start new plantations, as well as breeding programs in which some of the resistant progenies (F, FA, FB and FX) were crossed with highly productive clones of Asian origin (PB 86 and Tjir 1) [5]. Some of these resistant clones, such as the IAN and FX series, were planted commercially. Unfortunately, the resistance of these clones was broken by one of the *M*. *ulei* isolates [6]. The FX 3864 clone from the FX Series in the Vale do Ribeira area of Sao Paulo, Brazil, still shows good productivity and tolerance to SALB, unlike clones such as RRIM 600, PB 235, PB 86 and GT1. FX 3864 is the most widely grown clone in Colombia due to its productivity and tolerance to SALB. However, there are reports of increased susceptibility to *M*. *ulei* in the field [7] in some areas of the Colombian Orinoco region.

Our knowledge of the genes involved in the interaction of *H*. *brasiliensis* with *M*. *ulei* is very limited. Studies of differential gene expression during this interaction were reported by Garcia *et al*. [8], who studied the transcriptome of the MDF 180 clone, which is partially resistant to SALB, both in the field and in controlled conditions. Compared with the clone PB 314, which is considered highly susceptible to all *M*. *ulei* isolates, the resistance of the MDF 180 clone does not allow the formation of stroma and therefore impedes the completion of the fungal life cycle. The study was conducted by constructing subtractive libraries (SSH) at three different time points (6–72 hpi, 4–28 dpi, and 34–58 dpi) associated with the asexual and sexual development of the fungus after inoculation. Differentially expressed genes varied at different times. The identified Expressed Sequence Tags (ESTs) had an average size of 346 bp. The differentially expressed genes involved in the defense response of MDF 180 to *M*. *ulei* included pathogenesis-related (PR) proteins, R genes, proteins involved in the detoxification of reactive oxygen species (ROS) and phenol metabolism.

Studying the global transcriptome is important for understanding the molecular mechanisms associated with plant responses to pathogen attack, especially in non-model organisms [9]. It is now possible to sequence the entire transcriptome of an organism under a given condition using next-generation RNA sequencing technologies. This technology has increased the speed of gene, metabolic pathway and polymorphism discovery at a relatively low cost compared with earlier technologies such as SAGE, cDNA-AFLP, SSH and microarrays [10–13]. In the present study, sequences identified using RNA-Seq technology were analyzed according to two strategies: *de novo* assembly and reference-based assembly [9, 14]. Global transcriptomes were generated for the *H*. *brasiliensis* FX 3864 clone during the interaction with the *M*. *ulei* isolate GCL012. The aim of our study was to identify and functionally annotate the differentially expressed genes associated with the defense responses of *Hevea*, thereby improving our understanding of the molecular mechanisms underlying the tolerance of the rubber tree to biotic stress caused by SALB.

## Methodology

### *Hevea brasiliensis* plant material

Seedlings of the clones FX 3864 and RRIM 600 were provided by the company Mavalle S.A. located in the Colombian Orinoquia. FX 3864 is resistant and RRIM 600 is susceptible to the *M*. *ulei* isolate GCL012. The seedlings were transplanted into polyethylene bags that measured 25 cm wide and 35 cm long. The seedlings were kept for an adaptation period of 6 months, and soil fertilization was performed by applying Triple 15 every three months and Sephu-Amin foliar once a month.

Identification of the rubber clones was performed using microsatellites. DNA was extracted from leaf tissue [15] and PCR amplified using the primers SSRHb403 and SSRHb358, which were derived from the GenBank sequences AY486754 and AY486707. The PCR reactions were performed according to Garcia et al, 2011 [7]. DNA extracted from plant material from the Michelin Tres Pancadas plantation, located in the state of Bahia, Brazil, was used as reference standard.

### Isolation of *Microcyclus ulei*

The GCL012 isolate was obtained from leaflets of the GT1 clone located in the experimental farm plot of Corpoica-La Libertad Colombian Orinoquia. To obtain recent stromatic structures cultures, these isolates were cultured in M3 medium [16] and incubated at 25°C for 45 d in the dark stromas were subsequently macerated in 2 ml Eppendorf tubes with 1 ml of sterile distilled water and inoculated into 250 ml flasks with 50 ml of M4 sporulation culture medium (PDA, amino acids and peptone) [16]. Cultures were incubated for 12 d at 25°C in the dark and then exposed to white light for 90 min on days 13 to 15 [17]. Conidia were extracted from the medium using a solution of 0.05% Tween 80 and adjusted to a concentration of 1x10^5^ conidia/ml [18].

### Infection assays

Infection assays were performed under controlled conditions in cubicles of 2x1x0.8 m with metal support and coated with a black plastic, light and water curtain. Temperature were maintained between 22°C and 26°C, with a relative humidity above 85% and a photoperiod of 12 h light alternating with 12 h of darkness. *M*. *ulei* conidia were inoculated with a concentration of 1x10^5^ conidia/ml on Stage B leaflets using an airbrush [4]. Leaf tissue samples were obtained from the FX 3864 clone at 0 and 48 hpi [19]. The susceptible clone RRIM 600 was used as an infection control. For the viability control, conidia were inoculated with the airbrush on a plate containing culture medium M3.

### Total RNA extraction

A total of 0.2 g of leaflets was collected in 2 ml Eppendorf tubes at 0 and 48 hpi. Mechanical disruption was performed with quartz spheres in a mini bead beater (Biospec Products). One milliliter of extraction buffer (0.18 M Tris-HCl, 0.09 M LiCl, 4.5 mM EDTA, 1% SDS, pH 8.2) was added to the sample, followed by 300 μl of phenol equilibrated with TLE (0.2 M Tris-HCl, 0.1 M LiCl, 5.0 mM EDTA, pH 8.2), and the solution was mixed for 1 min by inversion and incubated at 50°C for 20 min. The sample was then centrifuged at 13,000 rpm for 20 min at 4°C. The aqueous phase was separated and transferred to a clean, sterile tube. Deproteinization was performed using three rounds of phenol-chloroform extraction, and the RNA was precipitated with one volume of isopropanol and incubated at -20°C for 12 h. The precipitated RNA was centrifuged at 13,000 rpm and washed three times with 500 μl of 70% ethanol. The resulting RNA was solubilized in 50 μl of RNase-free water [20]. Purification was performed using mini RNeasy columns (Qiagen Inc., CA, USA). Total RNA was quantified by fluorometry using Qubit, and total RNA quality was verified using an Agilent Bioanalyzer (Agilent Technologies, Mississauga, Canada).

### Library construction and sequencing

cDNA library construction and sequencing were performed by the Biotechnology Center of the University of Wisconsin. cDNA libraries were constructed from four RNA samples extracted from leaf tissue at 0 and 48 hpi using the RNA TruSeq V3 sample preparation kit (Illumina, Inc.). mRNA was isolated using oligo (dT) and fragmented using fragmentation buffer 5X. Reverse transcriptase and random primers were used to perform second strand-synthesis. The ends were then repaired for ligation of the primers. The products were purified and enriched with a PCR generating clusters with the final cDNA library. Samples were sequenced with paired ends (2x100 bp) on a multiplexed lane HiSeq2000 (Illumina, Inc.).

### Data availability

Short raw data reads were deposited at the Sequence Read Archive (SRA) (http://www.ncbi.nlm.nih.gov/) under the accession number (Bioproject Accession: PRJNA259872). Both *de novo* and reference-based assemblies can be obtained from the corresponding author.

### Preprocessing and transcriptome assembly

Quality control of the raw data streams was performed with FastQC 0.10.1 [21] using a minimum quality threshold Q20. The pre-processing tool FASTX-Toolkit 0.0.13.2 [22] was used to remove adapters, trimmed bases by position and clean artifacts. The Ribopicker 0.4.3 tool was used to identify and remove ribosomal contaminants [23].

The *de novo* assembly was performed with the software package Trinity released on 2013/02/25 [24]. The four samples, two from 0 hpi and two from 48 hours hpi, were assembled and analyzed by pulling all the reads from the sequencing into a single dataset. Trinity created a *De Bruijn* graph for each group of sequences and joined alternative isoform sequences and paralogous genes. Finally, transcripts supported in the original reads [24] were reconstructed. It was executed on paired-end reads and low-coverage *K-mers* that were not removed in the pre-processing were discarded. Default parameters of *K-mer* 25, minimum length of contig 200 and fragment length 500 were used. Runs with 12 CPUs and 80 G of memory were used for the *K-mers* count [24].

The quality of the *de novo* transcriptome assembly was evaluated by examining the number of both full- and partial-length transcripts assembled. For this analysis, the data were compared with the EST and Transcriptome Shotgun assembly (TSA) databases, as well as the *H*. *brasiliensis* genome locally downloaded from NCBI. Comparisons were carried out in the command line using BLASTn, megablast task, and max_target_seqs 1 e-value 1e-20. Then, the Trinity script analyze_blastPlus_topHit_coverage.pl was used to determine the coverage length of the transcripts. Additionally, *de novo* assembly contigs were aligned to the reference genome [25] using the same BLASTn parameters described in this paragraph.

For reference-based assembly of the transcriptome, scaffolds of the *H*. *brasiliensis* draft genome sequence (GenBank AJJZ00000000), accessed on 2013/03/15, were used. A *H*. *brasiliensis* genome index was constructed from 18.026 scaffolds using the Bowtie v2.1.0 software [26]. RNA-Seq reads at 0 and 48 h were aligned independently with their respective biological replicates to the reference genome, taking into account that paired-end reads were used for each sample. The software TopHat v2.0.9 [27] was used to overlap the reads separated by introns. Alignments were used to quantify the expression of genes and transcripts, taking into account that the number of reads produced for a transcript is proportional to its abundance. Transcript assembly was performed by overlapping the reads corresponding to each transcript using the default parameters in the software Cufflinks v2.1.1 [28]. Assemblies were performed individually for each sample. The consensus assembly of the transcripts was performed using the cuffmerge command for global transcriptome and the position or locus of the gene and isoforms in the genome. Additionally the *de novo* transcriptome was compared with the reference-based assembly transcriptome to confirm consistency. BLAST was used for this analysis, using BLASTn, megablast, and max_target_seqs 1 e-value 1e-20 as the execution parameters. The deduplication sequences was performed with dedupe.sh from BBMAP v34.56 tool using command line dedupe.sh in = file1.fasta,file2.fasta out = merge.fa.

### Estimation of transcript abundance and differential expression

The quantification of transcripts for the reference-based assembly was performed using the software RSEM 1.2.7 [29]. Reads were aligned to the transcripts obtained using Bowtie [30]. To estimate the abundance of each sample at 0 and 48 hpi, the expression-normalizing value FPKM (fragments per kilobase of exon fragments per million mapped) was calculated using the software RSEM 1.2.7. Gene and isoform counts from each samples were merged into a single matrix [29].

The software EdgeR 2.14 [31], written in R v2.15.3, was used to analyze the differential expression of the assembled transcripts. Files with all the read data in the abundance matrix were used taking into account the biological replicates at 0 and 48 hpi and using the default parameters. A TMM (Trimmed Mean of *M*-values) comparison was performed to compare transcript expression levels across samples. For this analysis, the length values of one of the biological replicates were extracted (48 h) and linked with the counts matrix to be analyzed. A new matrix with normalized expression values across samples measured in FPKM was then generated. The expression patterns of the transcripts in the samples were restricted to the transcripts with significant differential expression (P-value 0.01, Fold change log_2_ scale) at 0 and 48 hpi [32]. These were subsequently mapped to differential expression data of *Ricinus communis* using the Mapman v3.5.1R2 tool. [33]. Finally, the data were divided into a series of transcripts using the software R and grouped again according to common expression patterns using the script clusters_by_cutting_tree.pl—Ptree 20-R diffExpr.P0.001_C2.matrix.R.all.Rdata [32].

### Functional annotation

The assembled transcriptome and differentially expressed transcripts were subject to an analysis of similarity against non-redundant (nr) NCBI database accessed on 02/02/2014 and downloaded locally. An alignment was performed with the sequences from the transcriptomes and differentially expressed genes using the BLAST v2.2.27+ software [34] and the BLASTx algorithm with an E-value ≤ 10^−5^ and a high scoring segment pairs (HSP) cutoff limit at 33. The best alignment for each transcript was recovered by-max_target_seqs parameter 1. BLAST output in XML format was used for functional annotation. For this analysis, the Blast2GO software, written in java and using the command line B2G4pipe v2.5.0 [35], was used to recover gene ontology (GO) terms describing biological processes, molecular functions and cellular components. Annotations were refined with ANNEX and GOslim parameters for specific GO terms [36]. The software also used the KEGG (Kyoto Encyclopedia of Genes and Genomes) database, through which sequences were associated with enzymatic codes (EC: Enzyme commission) and the metabolic pathways in which they participate.

## Results and Discussion

**Fig 1:**
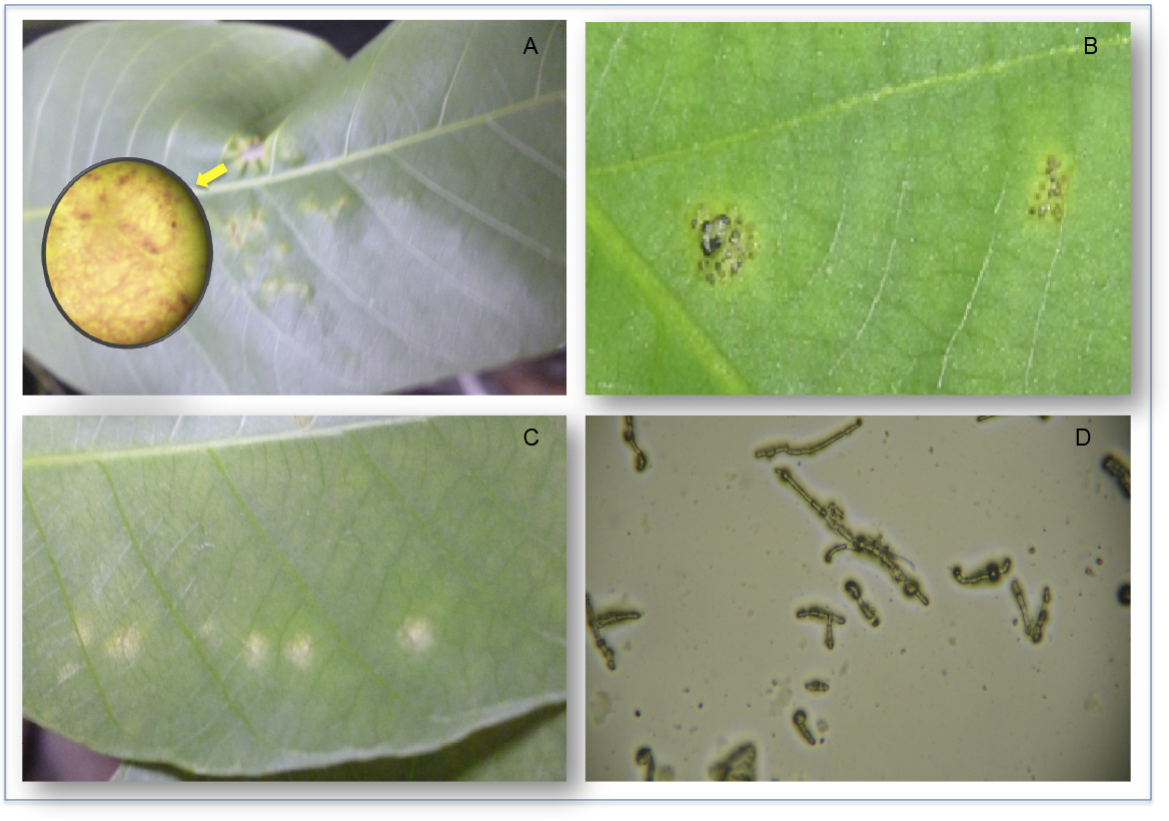
Signs and symptoms caused by *M*. *ulei* in *H*. *brasiliensis* leaflets. (A) Leaf deformation and sporulation of the GCL012 isolate 7 d post inoculation in RRIM 600. (B) Leaf showing the formation of stromatic structures of the isolate GCL012 at 24 dpi on RRIM 600. (C) A leaf of the clone FX 3864 showing the formation of chlorotic areas at 10 d post inoculation. (D) Germinating conidia of the GCL012 isolate. The germ tube can be observed (450X).

The identity of the clones FX 3864 and RRIM 600 was confirmed by the electrophoretic profile obtained with the microsatellites AY486754 and AY486707, which matched the reference standards (S1 Fig). Upon infection with the GCL012 isolate, the susceptible clone RRIM 600 showed symptoms of leaf deformation 7 d after inoculation (Fig 1A), whereas the resistant clone FX 3864 showed no signs of sporulation or foliar symptoms at 7 d post inoculation. At 24 d post inoculation, the clone RRIM 600 showed macroscopic signs, with the formation of *M*. *ulei* stromas on the leaf surface (Fig 1B), confirming the establishment of infection and the presence of the fungus on the plant. At 10 d post inoculation, chlorotic spots were observed for the clone FX 3864 (Fig 1C) at the sites where the leaves were inoculated. These spots could be due to the response of the plant to isolate the pathogen at the site of infection. After direct contact and penetration of the hyphae on the surface of the leaf, the cells of resistant clones collapsed due to a hypersensitive response (HR), which generated local yellowing of the leaf tissue. This HR induces the production of plant defense compounds such as phytoalexins, the formation of reactive oxygen species and the synthesis of callose for wall reinforcement, which eventually leads to cell death and restricts the site of infection [4]. In the viability control, conidia germination was observed after 24 h (Fig 1D).

The 48 hpi sampling time for the interaction between *Hevea sp*. and *M*. *ulei* was selected according to Lieberei (2007), who described the development of infection at a physiological level. This author showed that the fungus colonizes the intercellular space between 24 and 96 hpi. This host-parasite interaction activates *Hevea* gene regulation in response to *M*. *ulei* attack. Previous studies by our group evaluated the differential expression of genes from RRIM 600, PB 260, FX 3864, FX 2261 and IAN 710 clones against *M*. *ulei* by cDNA-AFLP validated by qPCR. This study showed greater variation in expression levels at 24 and 48 hpi [19].

### Computational analysis

#### Reads cleaning (pre-processing)

A total of 212,664,710 raw reads with paired ends were obtained for the four cDNA libraries sequenced. Each read had a length of 101 bp, resulting in a total of 40.5 Gb. After sequencing, short reads were demultiplexed and subjected to FastQC quality control [21]. This tool allowed the visualization of the quality of the sequencing data. At the same time, the FASTX-Toolkit was used to remove adapters, remove defective bases at the beginning and end of the reads, and filter low quality reads. This process increased the quality threshold per base for all samples above Q20, and reads lower than 20 bases were removed. A total of 191,046,408 reads were obtained after filtration, and rRNA sequences were removed using the Ribopicker v0.4.3 [23] tool. In total, 17.29% of the reads mapped to database sequences, mostly from ribosomal RNA. These sequences were removed from the dataset prior to assembly.

#### *De novo* transcriptome assembly

**Fig 2:**
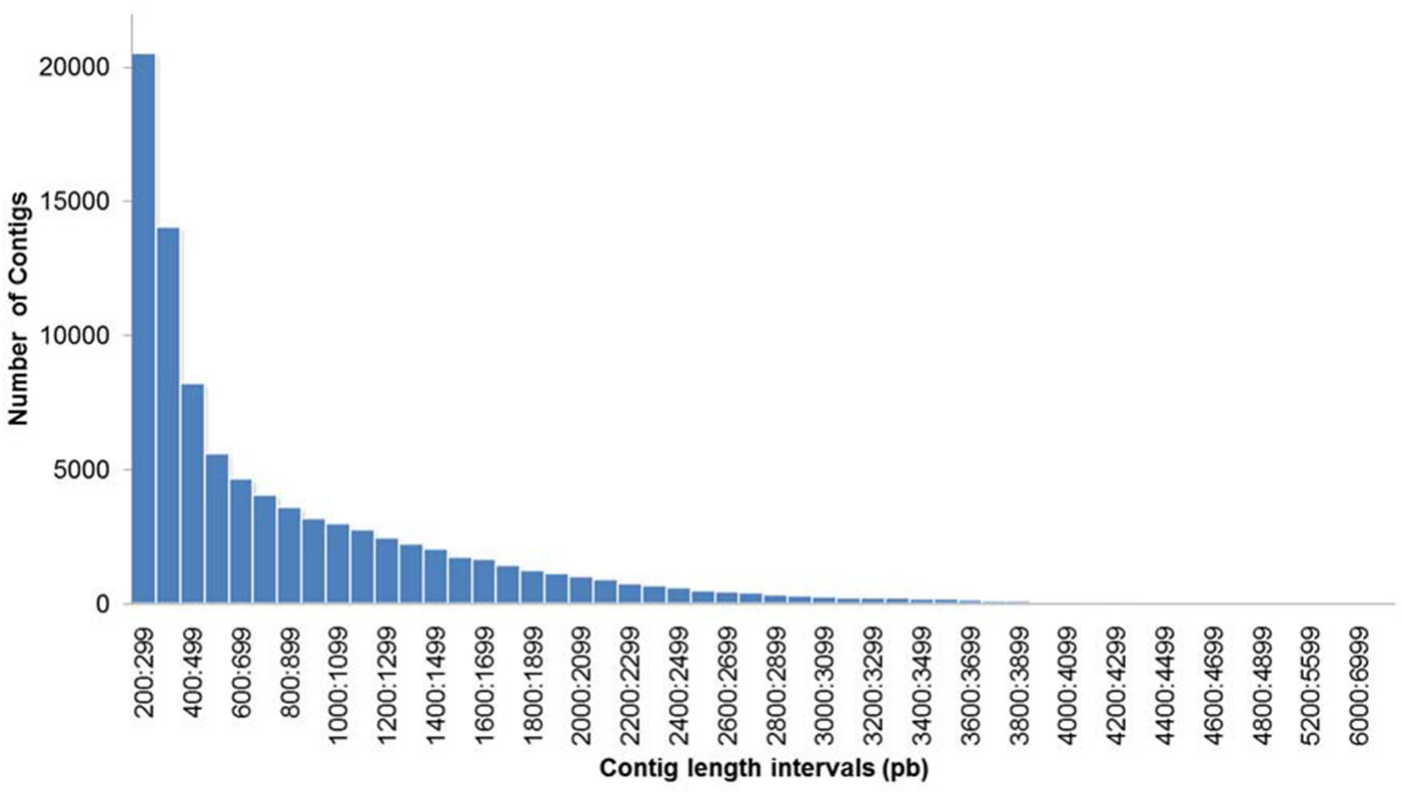
Distribution of contig lengths using Trinity *de novo* assembly for the *Hevea brasiliensis* transcriptome. All the contigs assembled are included.

A total of 158,134,220 paired-end reads for the four libraries were used for *de novo* assembly with Trinity. The algorithm first performed a greedily assembling, where a read was added iteratively to a read or contig until it was not possible anymore. Then, unique sequences were generated from reads forming sets of sequences to be overlapped [24]. The obtained contigs had a length ranging between 200 and 13,142 bp (Fig 2). A total of 132,312,332 reads, equivalent to 83.67% were correctly paired, and a total of 90,775 transcripts were obtained. Twenty duplicate sequences (0.02%) were removed from the assembly. Of these transcripts, 47,642 corresponded to genes with an N50 of 1.324, an average coverage of 24X, and an average length per transcript of 838 bp (Table 1). Similar results were obtained for other eukaryotic species such as *Cocos nucifera* using Illumina technology with short reads. A total of 54,931,406 reads were generated, and the *de novo* assembly program Trinity identified 57,304 genes with an average length of 752 bp [37]. In *Pogonus chalceus*, a total of 184,749,261 raw reads with paired ends were generated, and the assembly yielded a total of 65,766 contigs grouped in 39,333 isoforms (unigenes) with an N50 of 1904 [38].

**Table 1:** Summary data for sequencing and transcriptome assembly of *Hevea brasiliensis*. *De novo* and reference-based strategies.

Summary Data		All Samples
**Sequencing**	Raw reads (101 bp paired end)	212,664,710
	Bases (Gb)	40.5
	After cleaning reads	158,134,220
**Assembly**	***De novo* transcripts assembly**	90775
	Genes	47642
	% GC	40.87
	Contig N50	1324
	Max length (bp)	13142
	Min length (bp)	201
	Mean length (bp) (transcripts)	838
	Mean length (bp) (genes)	709
**(Bowtie)**	Read mappings (properly paired)	132,312,332
	Properly paired reads (%)	83.74
	Duplicate sequences	20
**Assembly**	**Reference-based transcripts assembly**	76278
	% GC	41.36
	Contig N50	1672
	Max length (bp)	12831
	Min length (bp)	201
	Mean length	1083
	Median	854
**Mapping**	Read mappings (properly paired)	132,330,507
**(Bowtie)**	Properly paired reads mean (%)	83.68
	Duplicate sequences	38

To evaluate the quality of the *de novo* transcriptome assembly, the 158,134,220 reads were aligned with Bowtie to the 90,755 transcripts generated after assembly. From these transcripts, 132,312,332 were correctly paired. A total of 25,687,668 reads (16.26%) corresponded to individual fragments with no overlaps (Table 1). This group of reads may correspond to potential repetitions, false positive, problematic reads (chimeric sequences) or small sequences. The evaluation of the consistency of the assembly with BLASTn (megablast) and an e-value of 1e-20 using transcript alignment to locally downloaded databases, showed that 51,621 transcripts were available on GenBank with the reference numbers GR305294.1-FE969198.1. A total of 48,024 transcripts (93%) matched the assembled transcriptome. Short cDNA sequences have a low quality due to their length, which varies between 500 and 800 nucleotides. However, these reads are very useful for measuring gene expression, annotating genes and for evaluating the consistency of the assembly [39]. The alignment of transcripts from the assembly with the 89,935 sequences available on TSA of *H*. *brasiliensis* GenBank: JT914190–JT981478 yielded 70,015 hits, equivalent to 77.15% of the sequences. The use of public databases allows the identification of known transcripts and the evaluation of assembly consistency and quality. The remaining sequences, corresponding to 7% of the sequences in the case of ESTs, can be due to new transcripts.

TSA sequences are the result of EST assembly with computational tools using mainly new-generation sequencing technologies with the objective of determining the transcriptome of an organism. This entails an accelerated pace of transcript discovery compared with previous technologies. Many transcriptional events are moment-specific or condition-specific. Thus, not all the genes are present in a specific transcriptome. Furthermore, sequences with assembly errors may appear. Therefore, some of the sequences of the transcriptome obtained in this study are not present in the TSA of *H*. *brasiliensis* [25].

The alignment of the transcripts assembled using the genome of *H*. *brasiliensis* with the GenBank accession number AJJZ00000000 showed that 89.44% of the 90,755 transcripts were found in the genome. The first draft of the *H*. *brasiliensis* genome, published in 2013, was used as a template to verify the quality of the transcripts assembled *de novo*. From a total of 90,755 transcripts aligned, 9,591 sequences equivalent to 10.56%, did not generate any hits. This result can be due to gene isoforms or to the fact that the genome is separated into scaffolds. Only 1.1 of the 2.15 Gb of reads were assembled, and 78% correspond to repetitive regions that can create assembly errors [25].

One of the advantages of *de novo* assembly is that no reference genome is necessary to obtain results from the original transcription. Furthermore, it is important to highlight that within these type of assemblies, the reads are not influenced by splicing, unlike reference-based assemblies, which may require prediction models [14]. The quality of the assembly also depends on the correct selection of the *K-mer*. This selection depends on sequencing depth, the read-error rate and transcriptome complexity [40, 41]. The *K-mer* selection for assembly was tested for different sizes ranging from K-19 to K-33. The K-25 default parameter of the Trinity software was finally selected, as it showed the best assembly statistics. The disadvantages of these types of assemblies are that they are very sensitive to sequencing errors and the presence of chimeric molecules in the data set. There is no effective way of distinguishing chimeric reads, from artifacts generated by the preparation of the library, from true reads [14]. Furthermore, the presence of repetitive sequences can hinder assembly. However, these regions are more common in introns than exons, and their presence can result in paired-end reads. These reads facilitate the reconstruction of original fragments encompassing the repeated fragments due to the way in which the adapters are placed.

#### Reference based assembly of the transcriptome (mapping)

**Fig 3:**
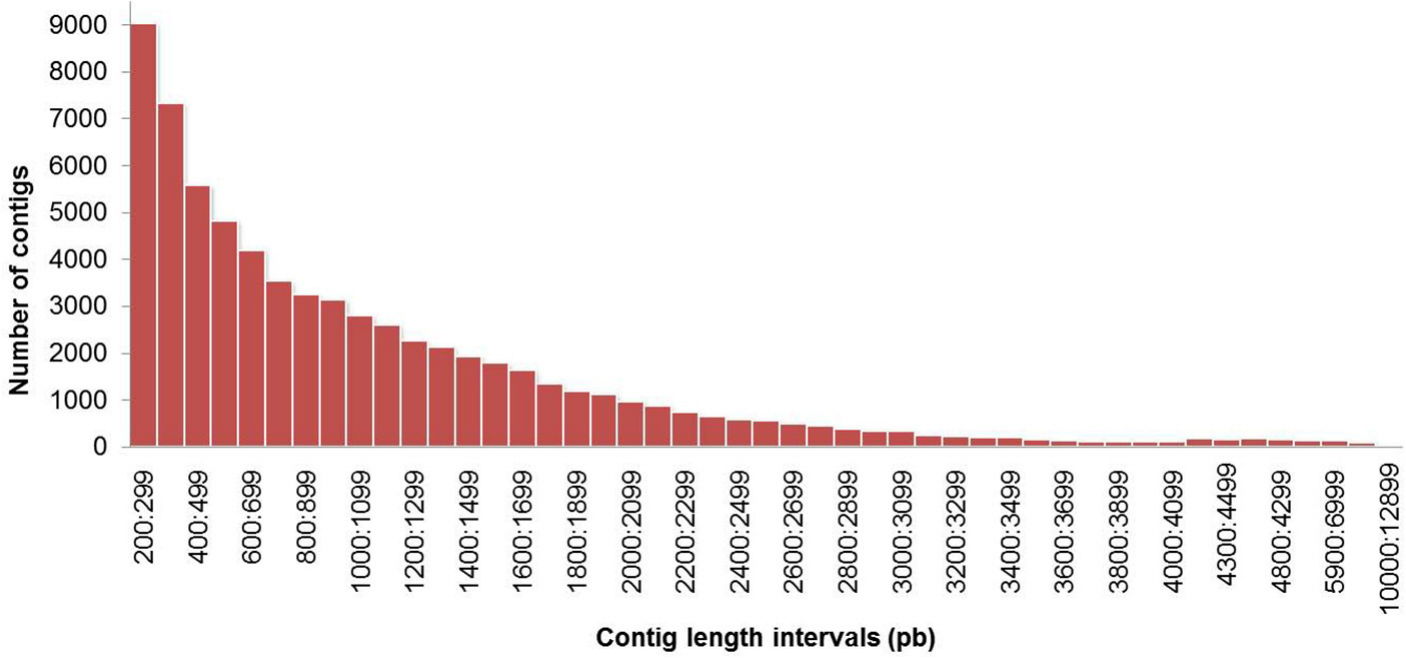
Length distribution of contigs obtained by using the TopHat-Cufflinks assembly (for reference) for the *Hevea brasiliensis* transcriptome. All contigs assembled were included.

The software TopHat [27] was used to align 158,134,220 paired-end reads to the 18,026 scaffolds of the genome of *H*. *brasiliensis*. This software mapped the reads to the reference genome and searched for splicing sites. The software Cufflinks [28] was then used to overlap the reads to each locus. The contigs obtained had an average size of 200 to 12,831 bp (Fig 3). The biological replicates of the samples from clone one and two were aligned individually to the genome of *H*. *brasiliensis*. A total of 132,330,507 were correctly paired with the mapping alignment. The failure of reads to map to the genome may be partly due to possible artifacts that have not been completely eliminated or because the genome present is not completely assembled. A total of 76,278 transcripts were recovered of which 38 (0.05%) duplicate sequences were removed from the assembly. Statistics showed an N50 of 1,672, a maximum length of 12,831, an average transcript length of 1,613 and a GC content of 41.36% (Table 1).

Reference-based assembly was computationally much faster than the *de novo* assembly because different loci in the genome served as templates for the independent alignment of reads at each position. Also, we discarded the presence of contaminating sequences or artifacts, as these did not align to the reference genome [14]. This strategy can also assemble low-abundance transcripts because of its high sensitivity. However, there are complex alternative splicing patterns in plants that are difficult to assemble accurately from short reads [14]. Furthermore, the reference annotation depends on the quality and assembly level of the annotated genome, that allow high sensitivity and accuracy to assemble full-length transcripts [42]. Moreover, the coverage necessary for a proper assembly and to obtain a catalog of well-represented transcripts may be approximately 10X, as opposed to *de novo* assembly, which requires approximately 30X coverage [14].

The comparison between the *de novo* and reference-based transcriptome assemblies by BLASTn (megablast) using an e-value-20 and max_target_seqs 1 showed that of the 90,755 transcripts assembled *de novo*, 15,722 sequences did not show reasonable hits against the 76,240 transcripts assembled by using the reference genome. This may be because the available *Hevea* genome is partially assembled (18,026 scaffolds) and contains a significant number of unresolved gaps. Many of these transcripts, witch were only present in the *de novo* assembly, could represent some of these unassembled sites.

The *de novo* assembly of transcripts showed low redundancy because only 20 duplicates were found (0.03%) and 159 contained sequences equivalent to 0.18%. Furthermore, in the reference-assembly we found 38 (0.05%) duplicates but a high number of contained sequences: 9,915 (13%). The low number of duplicates evidenced consistency on assemblies consensus obtained, because high redundancy can cause difficulties in the downstream analyses [43]. The high number of contained sequences in the reference-based transcriptome could be due to splicing-alternative events, that in plants are around 20%[44]. The consistency and length of the transcripts assembled by the reference strategy were superior to the *de novo* assembly (Table 1) because it was possible to obtain the complete sequences of the consensus transcripts found in the two assemblies. This process was useful for increasing the length of the *de novo* assembly transcripts.

#### Functional annotation of the transcriptome

A total of 90,750 transcripts presented BLAST hits. Five sequences were unsuccessful, possibly due to formatting errors or sequence length, and 28,167 were non-aligned singletons. The distribution of the species to which the sequences aligned according to putative functions can be found in (S2 Fig). A larger number of hits were found for plants of the species *Ricinus communis* (38%), *Populus trichocarpa* (23%), *Theobroma cacao* (8.3%) and *Vitis vinifera* (8.7%), among others. In total, 99% of the transcripts showed similarity with plant species. The most representative was the closely related species *Ricinus communis*, which belongs to the Euphorbiaceae family, whose genome has been completely sequenced [45].

**Fig 4:**
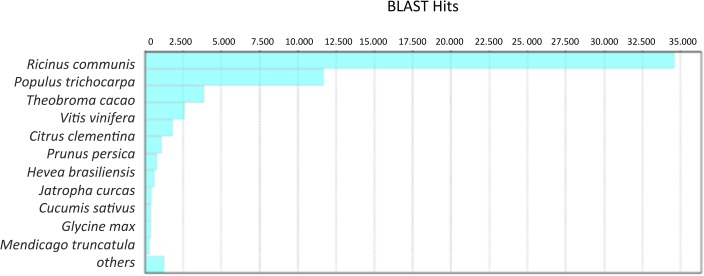
Species distribution (reference-based strategy). The results generated by sequence homology of transcripts against the NCBI nr database data.

The contigs obtained by reference-based assembly were analyzed in the same way as the contigs obtained in the *de novo* approach. Of the 76,240 transcripts, five sequences were not recognized due to formatting errors, sequence length or connection problems. A total of 14,441 singletons also did not correspond to the databases. *R*. *communis* (46%) also had the highest number of matches, followed by *P*. *trichocarpa* (15%), *T*. *cacao* (5.3%) and *V*. *vinifera* (3.3%), among others (Fig 4). The difference in the percentage of sequences assigned to each species between the two assemblies is due to the total number of transcripts obtained by each strategy. For *R*. *communis*, the sequence number was the same in both alignments, but this species was more represented in the reference-based assembly because the total number of transcripts was lower. The distribution of the five most represented species was similar for the two assemblies, which indirectly shows the quality of the transcriptomes obtained using the two strategies implemented. The 15,722 unique transcripts obtained by the *de novo* assembly were annotated independently. These showed the same trend species distribution (S3 Fig). Similar results were found in the global *H*. *brasiliensis* transcriptome assembly obtained from different tissues of clone RRIM 600, where the most representative species were *R*. *communis* and *P*. *trichocarpa* [46].

**Fig 5:**
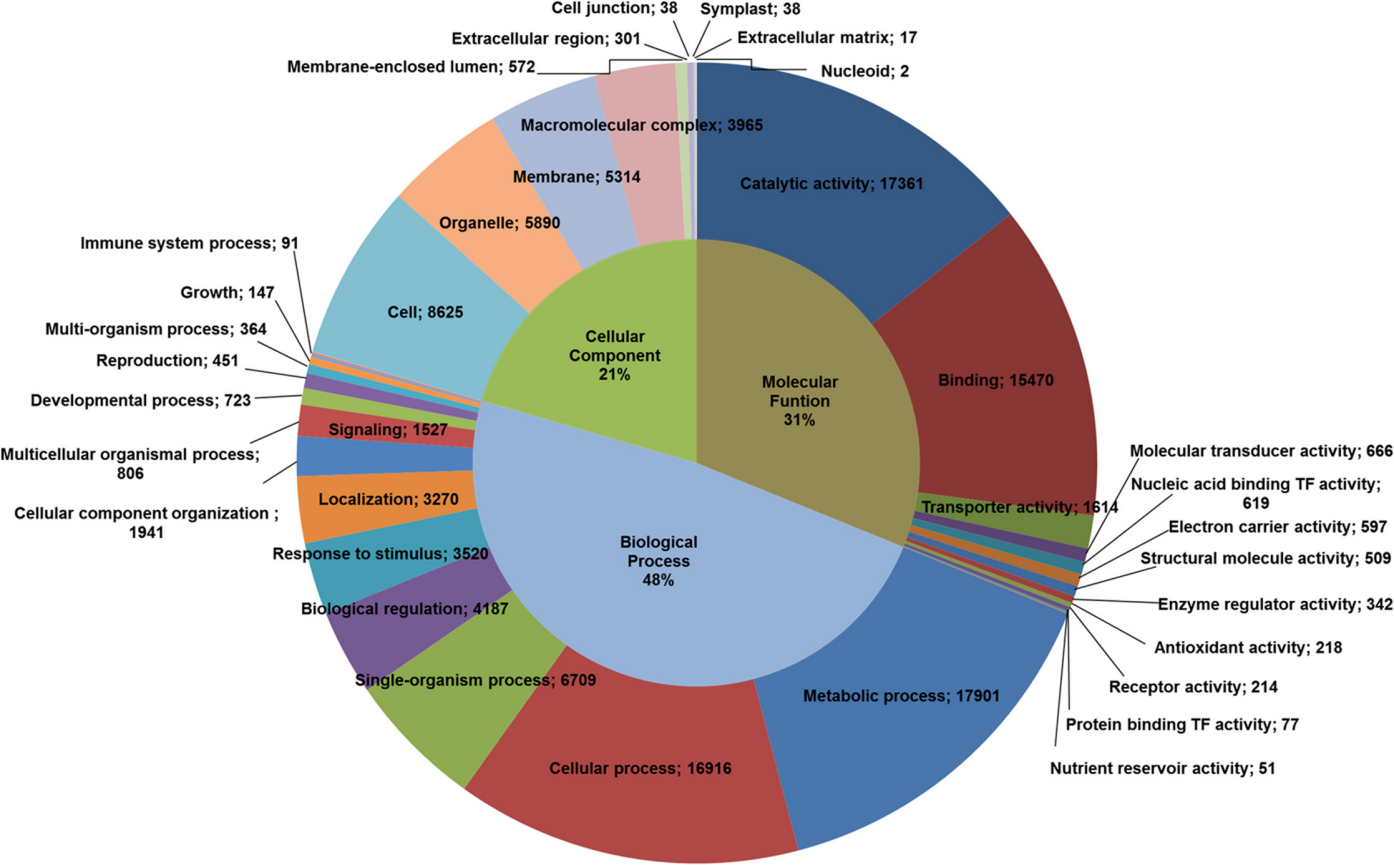
Distribution of GO terms from BLAST hits of the transcriptome of *Hevea brasiliensis* (reference-based assembly). Selected categories of GO terms are displayed at the top of the hierarchy for the biological process, molecular function and cellular component categories.

**Fig 6:**
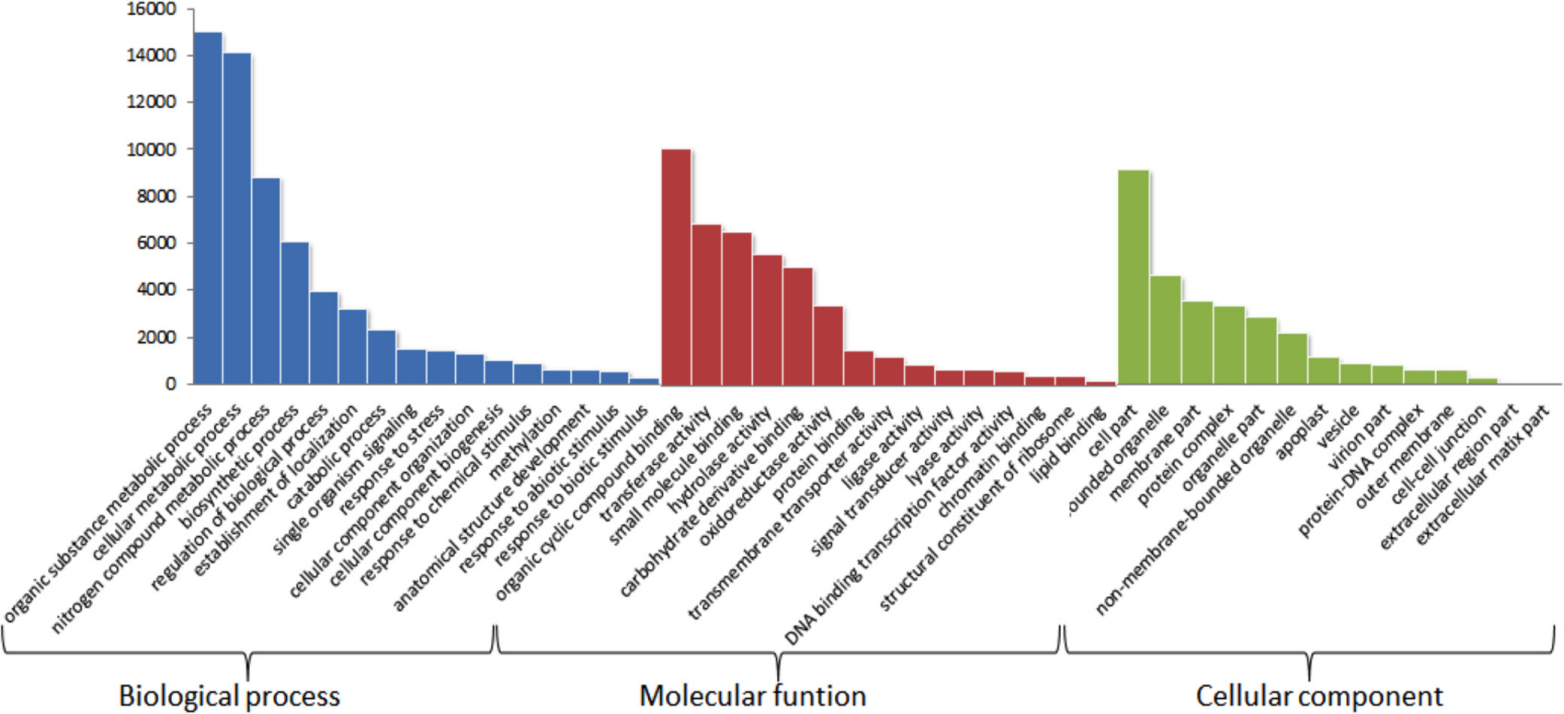
Distribution of GO terms from BLAST hits with the transcriptome of *Hevea brasiliensis* (reference-based assembly). The biological process, molecular function and cellular component (ontology level 3) categories are shown.

The software Blast2GO was used to annotate GO terms for both the *de novo* and reference-based assemblies. In the sequences from the *de novo* assembly, 127,568 terms were found and assigned to 31,035 transcripts. Of the three categories, 49% (58,437) of the terms associated with transcripts for biological processes (BP), 29% (39,559) to molecular functions (MF) and 22% (29,572) to cellular components (CC). Each category represents the highest level of hierarchical acyclic graph of the ontological terms found (S4 Fig). In the case of the reference-based assembly, a total of 121,058 terms were assigned to 29,185 transcripts. Of these terms, 48% (58,558) corresponded to BP, 31% (37,738) to MF and 21% (24,762) to CC (Fig 5). The main terms assigned to the three ontological categories were the general "catalytic activity", "metabolic processes" and "cell component". Similar results were reported by Salgado *et al*. (2014). In the MF category, "binding activity" and "catalytic activity" were the most represented. These categories are associated with high basal metabolic activity [47]. In the level-three ontologies, the most represented terms were "metabolic process of organic compounds" for BP, "binding to organic compounds" for MF and "cell components" for CC (Fig 6) (S5 Fig). The assignment of different ontological terms to the transcripts obtained by the two assembly strategies showed similar results, both in number of term assigned to each sequence and in the distribution of each category. This result suggests that either strategy can be used to obtain the global transcriptome in plants.

Blast2GO also allowed the identification of the EC numbers for both *de novo* and reference-based assemblies. A total of 14,417 ECs were assigned to 12,389 *de novo* transcriptome sequences involved in 139 metabolic pathways of the KEGG database. Similarly, 13,478 ECs were assigned to 11,791 transcriptome sequences from the reference-based assembly, and 137 metabolic pathways were associated. The major pathways involved were carbohydrate metabolism (sucrose and starch), nucleic acid metabolism (purines and pyrimidines), amino acids, energy generation and the production of secondary metabolites (Table 2). From 12 sub-metabolic pathway involved in natural rubber synthesis, we selected mevalonate pathway being this the most important [25]. Five enzymes were related in the annotation of the *de novo* assembly, these enzymes included acetyl-CoA, HMG-CoA reductase, mevalonate kinase, rubber cis-polyprenylcistransferase (CPT) and phosphomevalonate kinase, encoded in 16 sequences. In the reference-based assembly annotation, six enzymes were found corresponding to acetyl-CoA, HMG-CoA synthase, HMG-CoA reductase, mevalonate kinase, rubber CPT and mevalonate diphosphate carboxylase, which were encoded in 21 sequences. The enzymes found in the annotation by reference were found in a transcriptome annotation published by Salgado *et al*. [46], in this study they used different tissues to generate whole transcriptome assembly of RRIM 600 clone, through Roche/454 GS-FLXb (Titanium) technology. In addition to the five enzymes reported in the mevalonate biosynthetic pathway, the rubber CPT enzyme, which catalyzes the final reaction in the synthesis of natural rubber, was also identified, but it was not found by Salgado *et al*. [46]. These results revealed similarities between transcriptomes obtained from different sequencing technologies.

**Table 2:** Main specific metabolic pathways associated with the transcriptome of *Hevea brasiliensis* identified by KEGG.

Specific Metabolic Pathways	Reference-based assembly		*De novo* assembly	
	Number of Transcripts	Number of Enzymes	Number of Transcripts	Number of Enzymes
Starch and sucrose metabolism	656	34	550	36
Purine metabolism	575	46	724	51
Phenylalanine metabolism	279	18	270	20
Pyrimidine metabolism	275	27	436	25
Glycerolipid metabolism	214	17	190	17
Pyruvate metabolism	205	23	173	21
Cysteine and methionine metabolism	195	29	238	27
Glycolysis / Gluconeogenesis	193	23	173	24
Aminoacyl-tRNA biosynthesis	180	21	180	21
Flavonoid biosynthesis	179	13	182	13
Arginine and proline metabolism	173	32	211	35

#### Differential expression analysis

After conducting a comprehensive analysis of the transcriptomes from the two assembly strategies, a search was performed to identify differentially expressed genes that may be involved in the response of clone FX 3864 to infection by the *M*. *ulei* isolate GCL012. The reference-based transcriptome assembly was used for the differential expression analysis although the two different transcriptome assemblies showed similar annotations. Furthermore, 83% of the transcripts were obtained in both assemblies, and the transcript length was lower for the *de novo* assembly, with an N50 of 1,324 and an average length of 838 bp. For the reference-based assembly, the N50 was 1,672, with an average length of 1,083 bp.

For the analysis of differential expression profiles, reads were aligned to contigs assembled to quantify transcripts abundance. The gene expression data were normalized with FPKM between different RNA-Seq samples and used to compare the expression levels at 0 and 48 hpi. After the transcript abundance was quantified and normalized with the RSEM software, the EdgeR software was used to take into account the biological variability between replicates. Normalization was performed by default using a TMM scale (Trimmed Mean Values of M), which takes into account differences in sequencing depth between samples [48]. It is also important to mention that EdgeR is a method designed for experiments with low numbers of replicates, and thus was appropriate in the context of this project [48].

**Fig 7:**
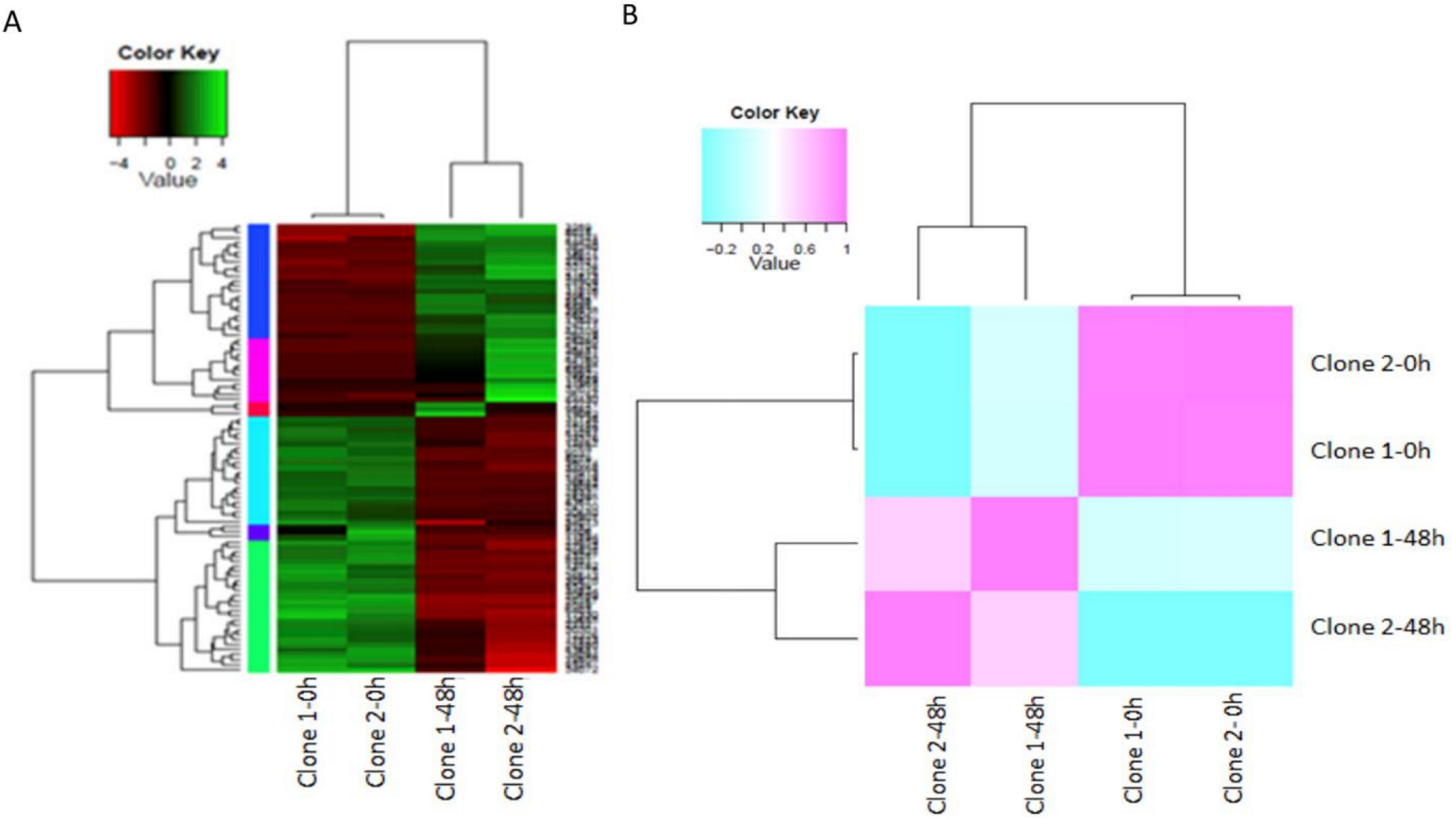
Comparison of transcription profiles between samples. (A) Heat map with hierarchical clustering of transcripts and samples. Significant differential expression values measured in FPKM with (P 0.01) are shown. The colored bars show the relative expression levels for each transcript (row) and each sample (column). (B) Hierarchical clustering using a Spearman correlation matrix resulting from the comparison of transcript expression values (FPKM normalized by TMM) for each pair of samples.

**Fig 8:**
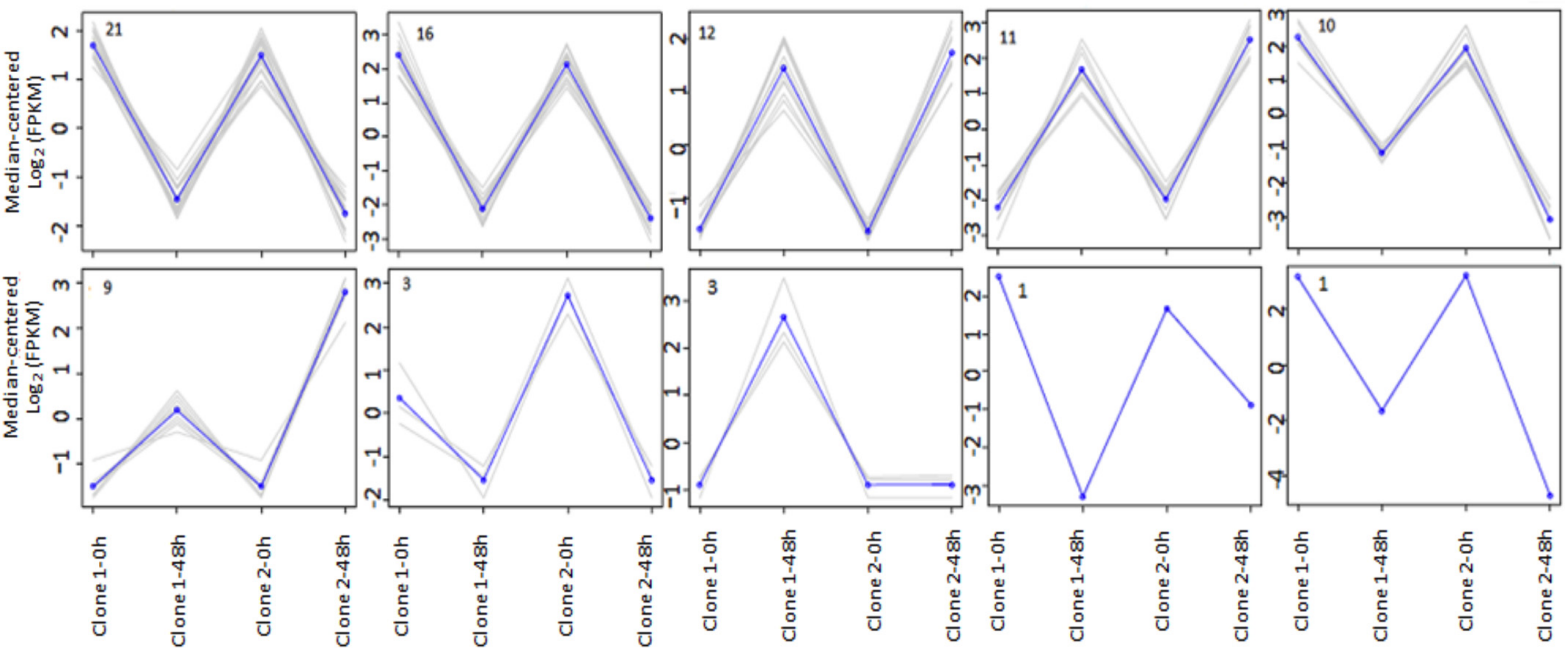
Groups with common expression profiles. Each graph corresponds to a hierarchical clustering of transcripts. X-axis: Clone 1 samples at 0 and 48 hpi, clone 2 at 0 and 48 hpi. Y-axis: centered median of log2 (FPKM). The blue line corresponds to the average expression values per group. Gray lines represent individual transcripts. The number of transcripts per group is shown in the upper left part of each graph.

The expression analysis compared the transcriptional profiles of the clone FX 3864 at 0 and 48 hpi, which were grouped hierarchically in a heat map. The measured expression values were transformed into log2 FPKM to obtain a centered median for each gene (Fig 7A). Comparisons of gene expression values measured by FPKM and normalized by TMM were performed for each pair of samples using a Spearman correlation matrix, where extreme values of 1 correspond to a high positive correlation when the samples of 0 and 48 h were compared with themselves or their biological replicates. Values of -0.2 were obtained when there was no correlation, such as when the times 0 and 48 hpi were compared (Fig 7B). A total of 35 genes were found to be up-regulated, and 51 were down-regulated. Each gene was grouped according to its behavior in common expression profiles (Fig 8).

#### Functional annotation of the differential expression

The ontological terms assigned to differentially expressed genes within the three categories showed that transcription regulation processes were the most represented for BP at ontology level 4, followed by signal transduction, stress responses and hormone responses. These results indicate that the modulation of the plant transcriptional responses was related to the stress caused by the interaction with the fungus *M*. *ulei* within the first 48 hpi. For FM, the most represented category was nucleic acid binding, followed by phosphotransferase activity and hydrolase activity (S6A Fig). The first category is related to a large number of transcription factors associated with the regulation of biotic stress responses, the second category is associated with signal transduction and the last category is associated with hydrolase activity during cell wall synthesis and remodeling (S6B Fig). Finally, in cellular component the category most representative was intracellular organelle. (S6C Fig).

A total of 86 genes were differentially expressed in the FX 3864 clone between 0 and 48 hpi with the *M*. *ulei* GCL012 isolate, based on a P-value of 0.01 (S1 Table). The differentially expressed genes had an average size of 2,294 bp. While assigning gene functions using Blast2GO, we observed similarities between the proteins of different plant species: 47 corresponded to *R*. *communis* Euphorbiaceae species closer to *Hevea sp*, 14 to *T*. *cacao*, 13 to *P*. *trichocarpa*, three to *Citrus siniensis*, three to *H*. *brasiliensis*, two to *Prunus mume*, one each to *V*. *vinifera*, *Manihot esculenta*, and *Fragaria vesc*a and yet one failed to find a hit with the bases used for annotation. Of the total differentially expressed genes, 37 were found to be up-regulated, which corresponded to 42%. The remaining genes were down-regulated.

Eleven transcripts corresponded to hypothetical proteins. Among the up-regulated genes, three were associated with the synthesis and remodeling of the plant cell wall, two glycosyl hydrolases from families 3 and 9 (Hb36621 and Hb44430) and a polygalacturonase (Hb42274). Glycosyl hydrolases family 3 (GH3) enzymes are widely distributed in bacteria, fungi, plants and animals, where they perform different activities such as exo-β-D-glucosidase, α-L-arabinofuranosidase, β-D-xylopyranosidase and N-acetyl-β-D-glucosaminidase (S1 Table) [49]. N-acetyl-β-D-glucosaminidase activity is associated with chitin degradation and has been widely described in bacterial GH3 enzymes but had not yet been described in plants [50].

Proteins involved in plant cell wall metabolism, such as glycosyl hydrolases from family 9, are associated with susceptibility to necrotrophic pathogens. Tomatoes with mutations in the genes endo-1,4-β-glucanase cel1 and cel2 showed resistance to *Botrytis cinerea* but were highly susceptible to *Pseudomonas syringae* pv., which is considered a biotrophic pathogen [51]. Furthermore, the production of polygalacturonases has been described as a strategy to trigger plant defense responses through homogalacturonan fragments (HG) derived from the degradation of the cell wall. These fragments have also been found to induce the accumulation of SA [52].

Histidine kinase (Hb31968) up-regulation is associated with signal transduction triggered by the detection of the pathogen. This kinase has a CHASE (Cyclases/Histidine kinases Associated Sensory Extracellular) transmembrane domain and a receptor with a kinase domain according to predictions made with HMMER [53], and it is considered to be an independent ethylene receptor. These types of proteins have been found to be up-regulated during biotic and abiotic stresses [54]. The transcript Hb320124, which is similar to a sensor domain of the histidine kinase signal transduction pathway, was also up-regulated. These two proteins are associated with the widely described bacterial two-component signaling systems, which are also found in *Arabidopsis* [54].

Transcript Hb5539, with homology to CPR-5, was identified among the underexpressed transcripts. This transmembrane protein is constitutive, related to pathogenicity and associated with the down-regulation of different transcription factors [55]. Studies in *Arabidopsis cpr5* mutants have demonstrated increased SA production, which confers resistance to the *Peronospora parasitica* [56]. The down-regulation of this protein could be linked to the increase of SA as a defense mechanism in *H*. *brasiliensis* against *M*. *ulei*.

Six transcripts were found to be associated with the JA/ET pathway. These transcripts contained apetala2/AP2 domains (Hb12462, Hb23559, Hb5424, Hb17330, Hb20167 and Hb34468), and the transcript Hb43251 contains additional RAV1 domain. All of these transcripts are classified within subgroup B, which is involved in plant defense responses. Group B is also sensitive to the concentration of different hormones such as ET, JA and SA. This suggests that the transcriptional regulation of the JA/ET pathway is mediated by the production of high levels of SA, which are characteristic of plant responses to attack by biotrophic or hemibiotrophic pathogens (S7 Fig) [57]. The AP2 domain superfamily is also called ERF (*Ethylene Response Factor*) due to the ET dependence of the response of these transcription factors [58].

The expression analysis of the *Arabidopsis* transgenic *35s*:*ERF1*, in which ERF1 is constitutively expressed, showed that genes associated with cell wall reinforcement, such as xyloglucan endotransglycosylase, were up-regulated [59]. This protein and ERF were down-regulated in the present study. However, the response regulators RR were found to be down-regulated in *35s*:*ERF1*. In this present study, histidine kinases with regulatory domains associated with two-component signaling were up-regulated. This observation corroborates the behavior observed with the other genes for which there are antagonistic behaviors between the JA/ET and SA pathways.

According to the expression profiles obtained at 48 hpi, this work suggests that the resistance of FX 3864 to *M*. *ulei* is mediated by the induction of SA-dependent defense genes. This result is inferred based on the suppression of the JA/ET pathways due to an increase in SA. In the ESTs recovered from the subtractive libraries of a study by Garcia *et al*. [8], five proteins associated with pathogenicity (PR1a, NtPRp27-like protein, Bet v I allergen, Beta1,3-glucanase and hevamine), four protease inhibitors and one pectinesterase inhibitor were found in clone MDF 180 compared with the susceptible clone PB 314. These proteins were associated with the inhibition of mycelium growth and were not present in the libraries of clone PB 314. A transcriptional study using cDNA-AFLP in clones FX 3864, FX 2261 and RRIM 600 inoculated with the GCL009 isolate showed 49 differentially expressed genes that were associated with R genes, transcription factors, protein degradation, sphingolipids and cell wall synthesis, together with a transcript may be associated with the virulence of the fungus [19]. The findings of these studies differ from ours most likely due to the *Hevea* clones, the *M*. *ulei* isolate and the techniques used. These reasons could explain the differences in the differentially expressed genes found in our study. However, all these studies help in understanding the molecular mechanisms involved in the interaction of *H*. *brasiliensis* with *M*. *ulei*.

### Conclusion

The similarity in both the statistical metrics of the assembly and the results of the functional annotation of transcripts obtained from the *de novo* and reference-based assembly strategies allows the implementation of either strategy to generate global transcriptomes from short reads. The use of the *H*. *brasiliensis* reference genome for assembly provided longer transcripts than those obtained by the *de novo* approach. This approach generates more complete gene sequences that facilitate further studies of the identified genes.

The ontological terms found in the differential expression analyses were mainly associated with signal transduction, responses to hormonal stimuli and responses to biotic stress. The results suggest that the modulation of the plant transcriptional responses was related to the stress caused by the interaction with the fungus *M*. *ulei* within the first 48 hpi. The down-regulation of seven ET-sensitive genes from the putative superfamily AP2/EFR allowed to infer a decrease in ET and JA within the first 48 hpi of the *H*. *brasiliensis*-*M*. *ulei* interaction. Additionally, the up-regulation of genes associated with cell wall metabolism, such as Hb42704 with homology to a polygalacturonase, and the down-regulation of Hb5539, which is a homologue of CPR-5, suggests an increase in SA within the first 48 hpi of interaction between the resistant clone FX 3864 and the *M*. *ulei* GCL012 isolate.

## Supporting Information

S1 Fig Electrophoretic profiles for varietal identification of clones FX3864 and RRIM-600. Example profiles were obtained from some individuals of each clone. Left: capillary electrophoresis, microsatellite primers SSRHb358 and primer AY486754. Right: 7% polyacrylamide gel electrophoresis, microsatellite SSRHb403 and primer AY486707. **M**. Molecular weight markers. **C**. Reference standards for each clone.
(TIF)

S2 Fig Distribution of species (*de novo* strategy). These results were generated based on transcripts sequence homology compared with NCBI nr database data.
(TIF)

S3 Fig Distribution of species unique transcripts (*de novo* strategy). These results were generated based on transcripts sequences not found in reference-based assembly homology, compared with NCBI nr database.
(TIF)

S4 Fig Distribution of gene ontology terms from BLAST hits with the transcriptome of *Hevea brasiliensis* (*de novo* assembly). Selected GO term categories are shown at the highest level of the hierarchy for the divisions of biological process, molecular function and cellular components.
(TIF)

S5 Fig Distribution of gene ontology terms from BLAST hits with the transcriptome of *Hevea brasiliensis* (*de novo* assembly). The categories biological process, molecular function and cellular component (ontology level 3) are shown.
(TIF)

S6 Fig Ontological terms associated with the differential gene expression during the *H*. *brasiliensis*-*M*. *ulei* interaction. Categories; (A) Biological process. (B) Molecular function. (C) Cellular component.
(TIF)

S7 Fig Expression data Mapman plot. Transcripts of *Hevea brasiliensis* associated to biotic stress from *Ricinus communis* (affimetrix arrays). From 86 differential expressed genes 74 were mapped (some of the data points may be mapped multiple times to different bins) and visible in this pathway: 30
(TIF)

S1 Appendix Differentially expressed transcripts. (TXT)

S1 Table Differentially expressed genes between 0 and 48 hpi. Putative sequences assigned by similarity.
(XLSX)
