# Supplementary figures and images for: Assembly and Analysis of Differential Transcriptome Responses of Hevea brasiliensis on Interaction with Microcyclus ulei
Source: PLoS One. 2015 Aug 19;10(8):e0134837. doi: 10.1371/journal.pone.0134837 (PMC4564276; doi:10.1371/journal.pone.0134837)

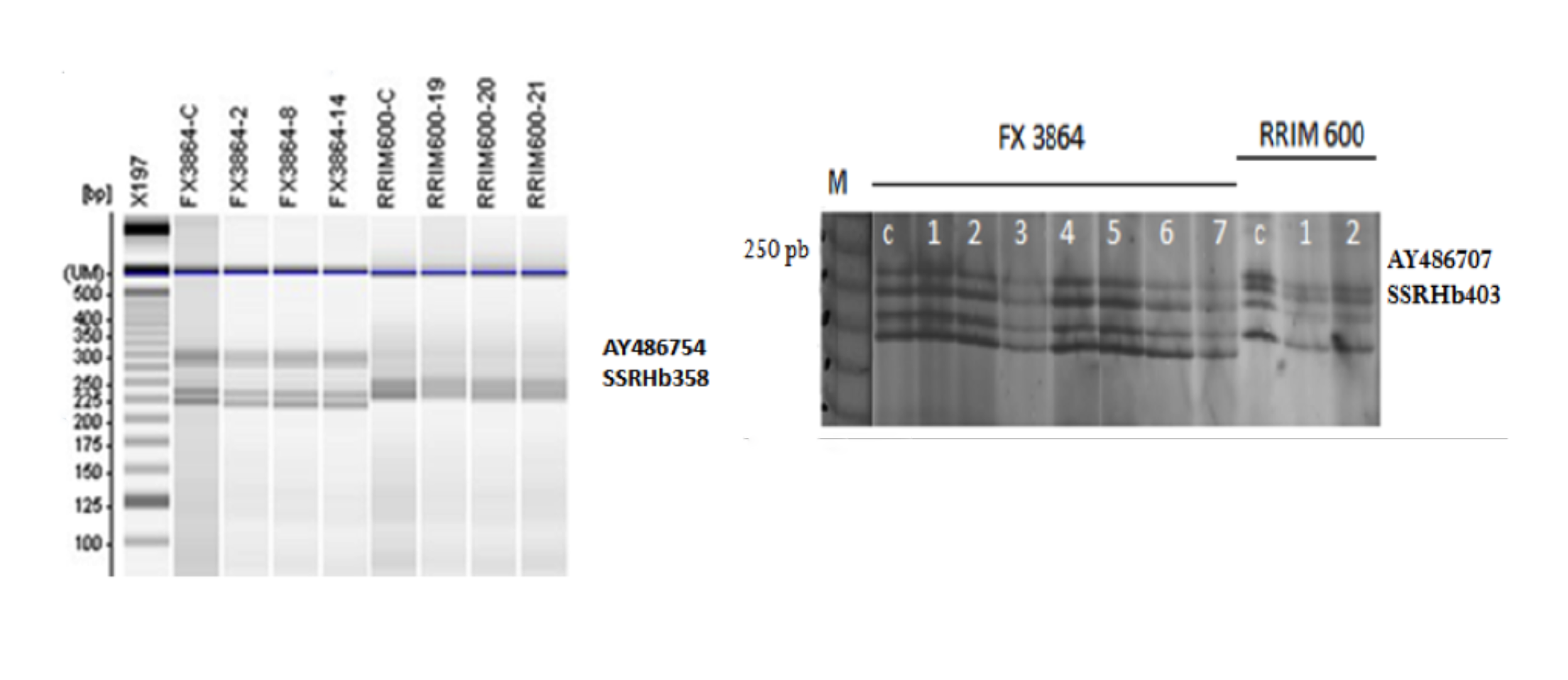

Supplement: S1 Fig — Example profiles were obtained from some individuals of each clone. Left: capillary electrophoresis, microsatellite primers SSRHb358 and primer AY486754. Right: 7% polyacrylamide gel electrophoresis, microsatellite SSRHb403 and primer AY486707. M. Molecular weight markers. C. Reference standards for each clone. (TIF) [file pone.0134837.s001.tif]

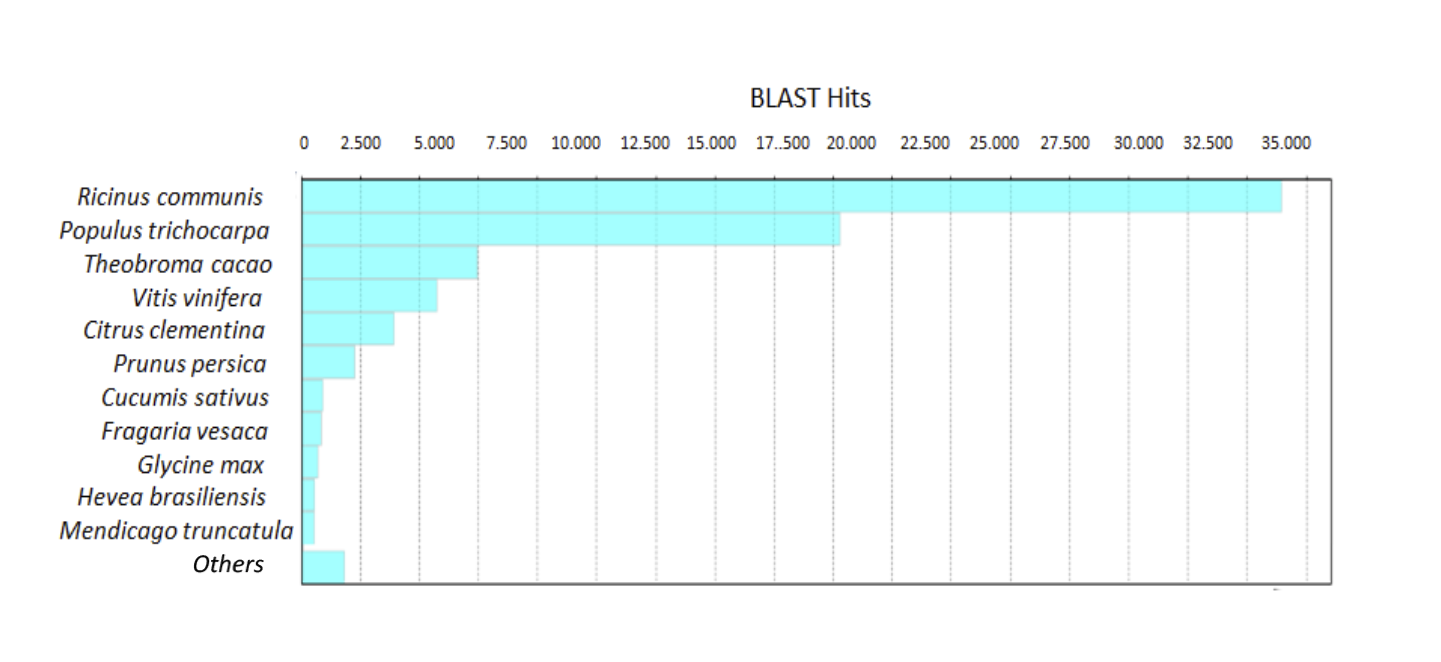

Supplement: S2 Fig — These results were generated based on transcripts sequence homology compared with NCBI nr database data. (TIF) [file pone.0134837.s002.tif]

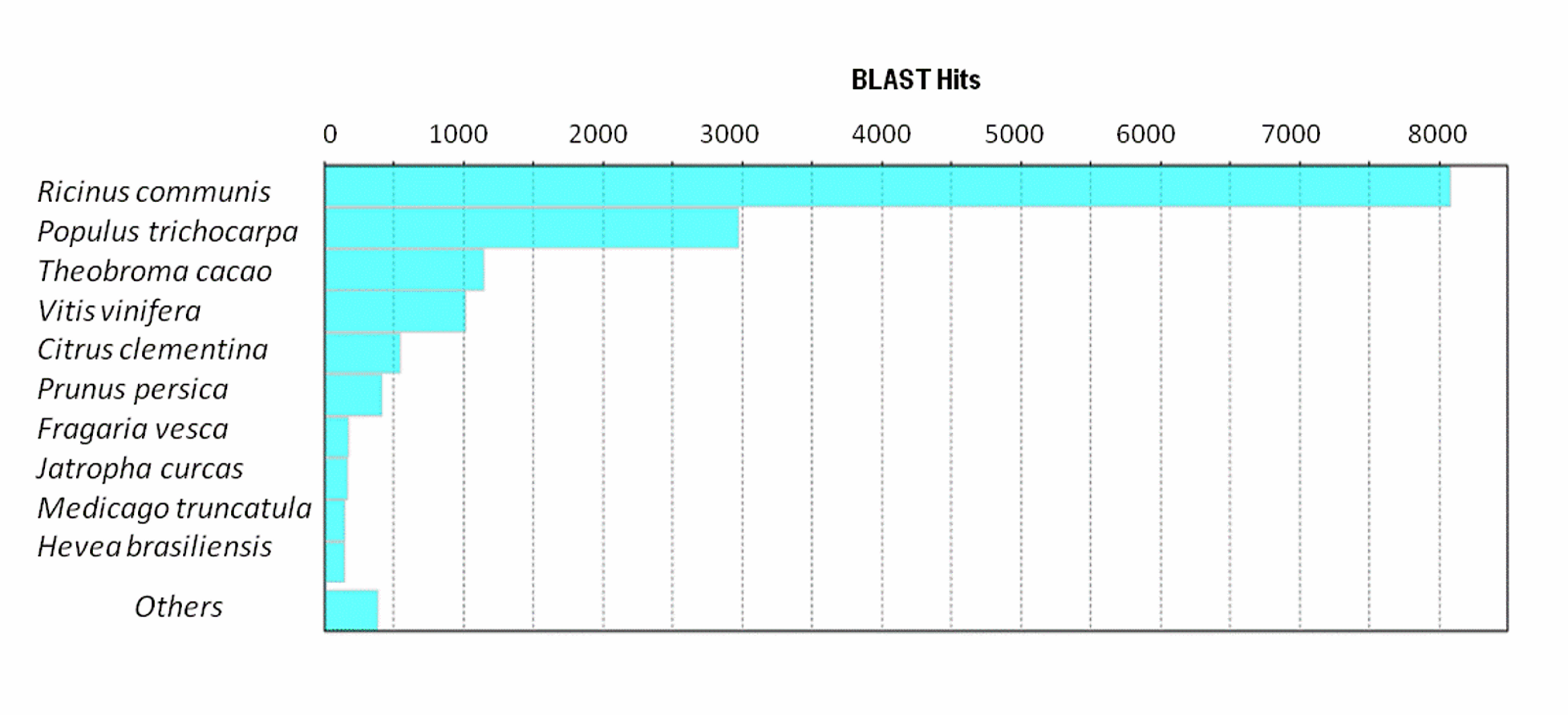

Supplement: S3 Fig — These results were generated based on transcripts sequences not found in reference-based assembly homology, compared with NCBI nr database. (TIF) [file pone.0134837.s003.tif]

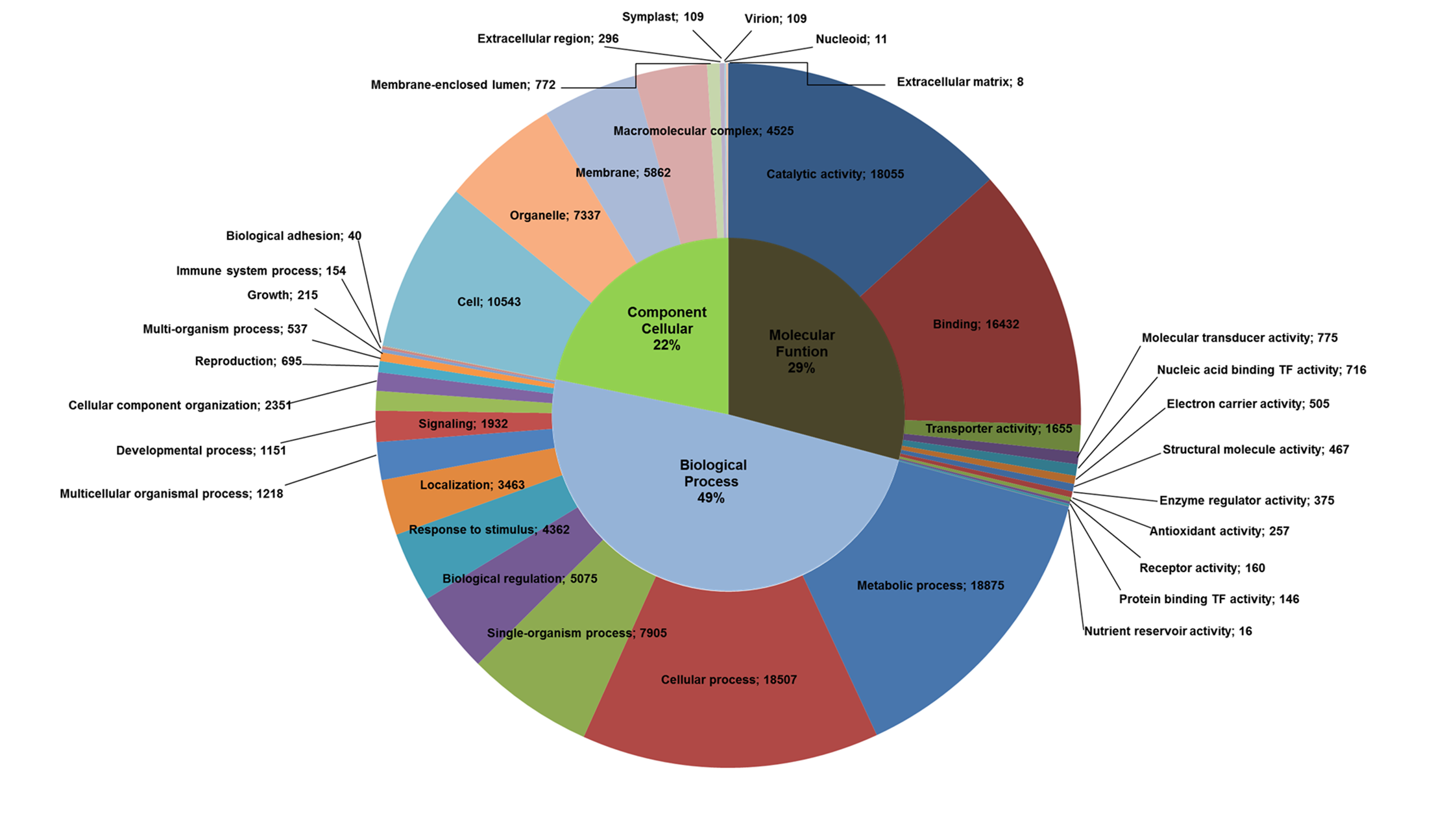

Supplement: S4 Fig — Selected GO term categories are shown at the highest level of the hierarchy for the divisions of biological process, molecular function and cellular components. (TIF) [file pone.0134837.s004.tif]

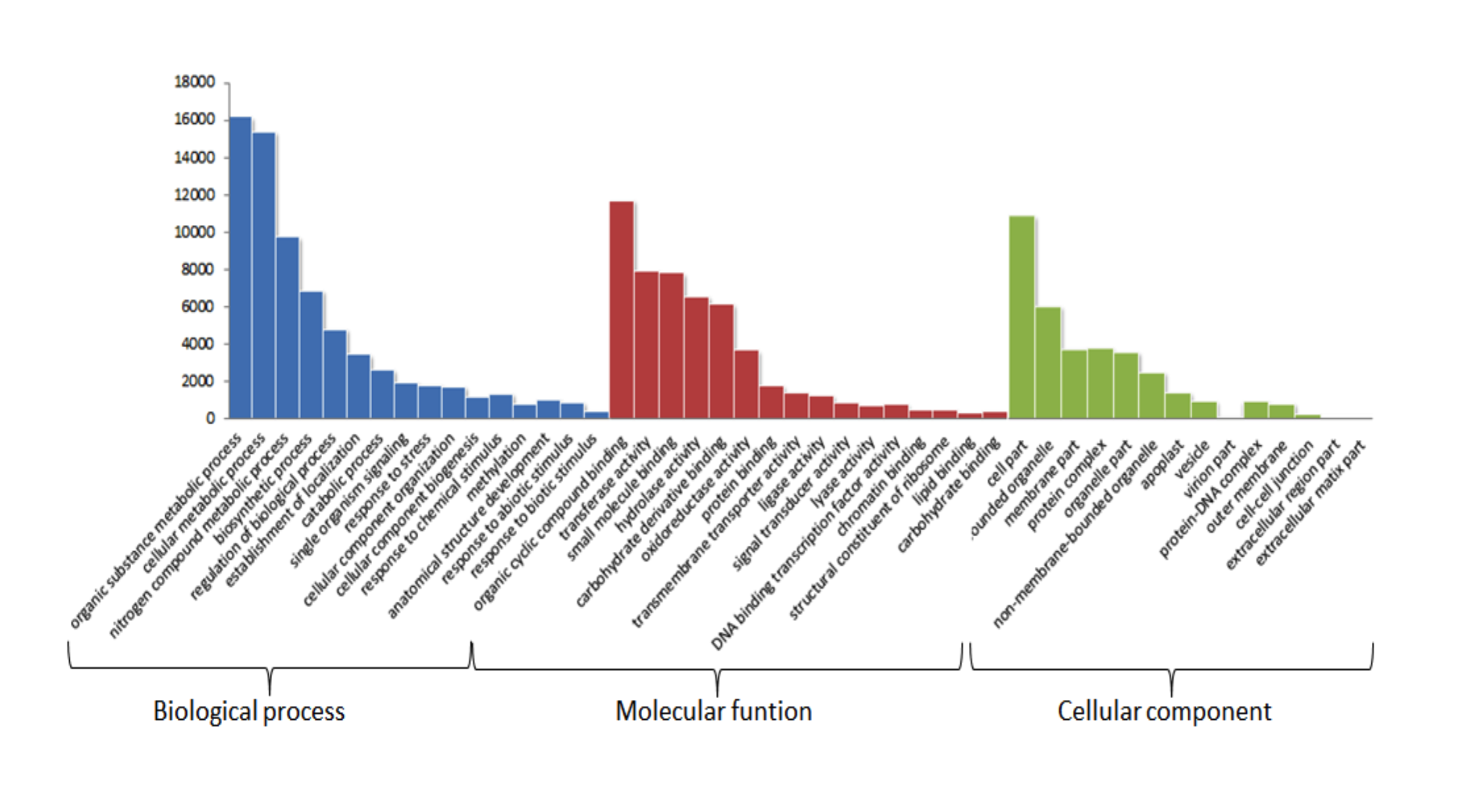

Supplement: S5 Fig — The categories biological process, molecular function and cellular component (ontology level 3) are shown. (TIF) [file pone.0134837.s005.tif]

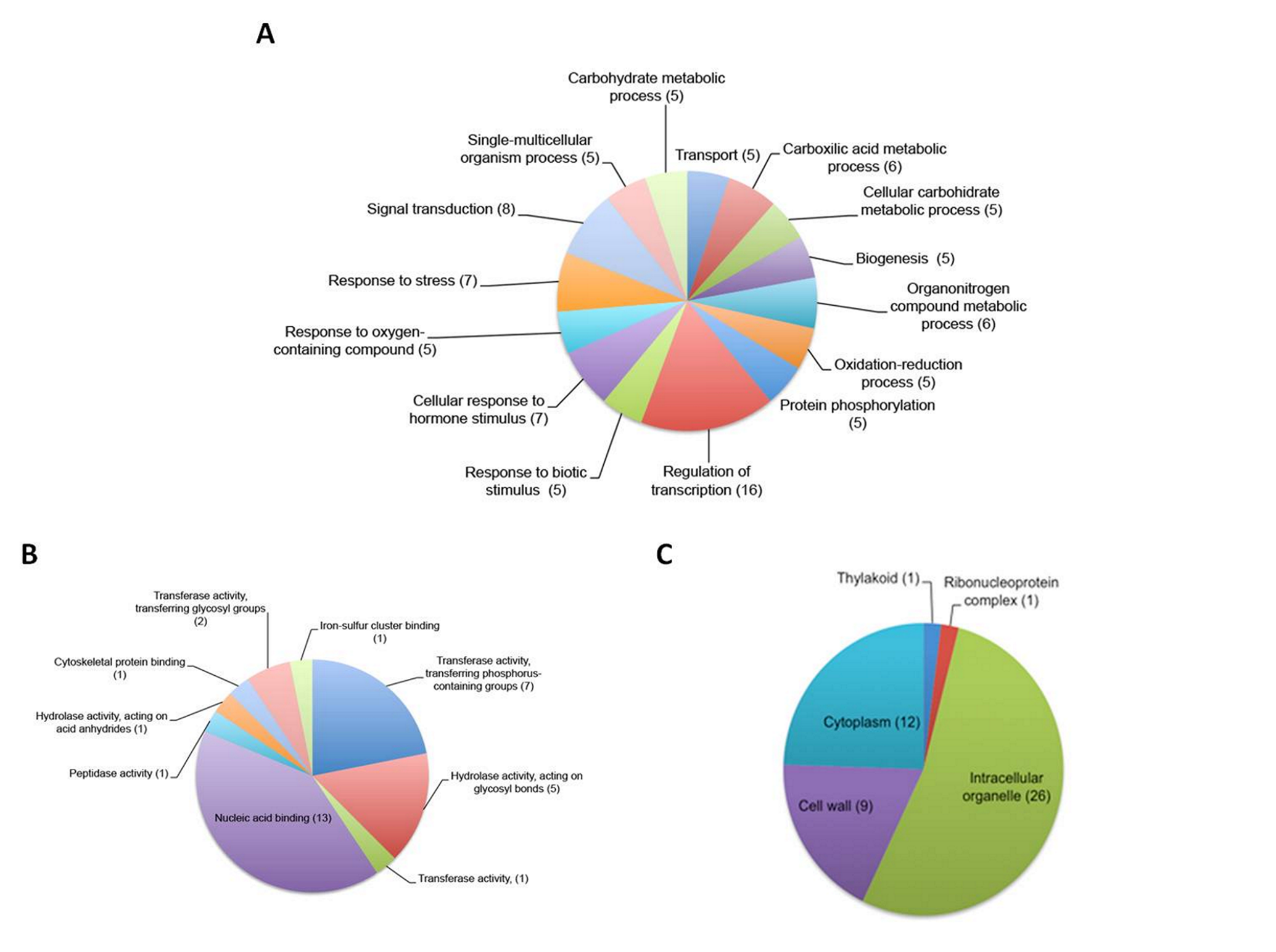

Supplement: S6 Fig — Categories; (A) Biological process. (B) Molecular function. (C) Cellular component. (TIF) [file pone.0134837.s006.tif]

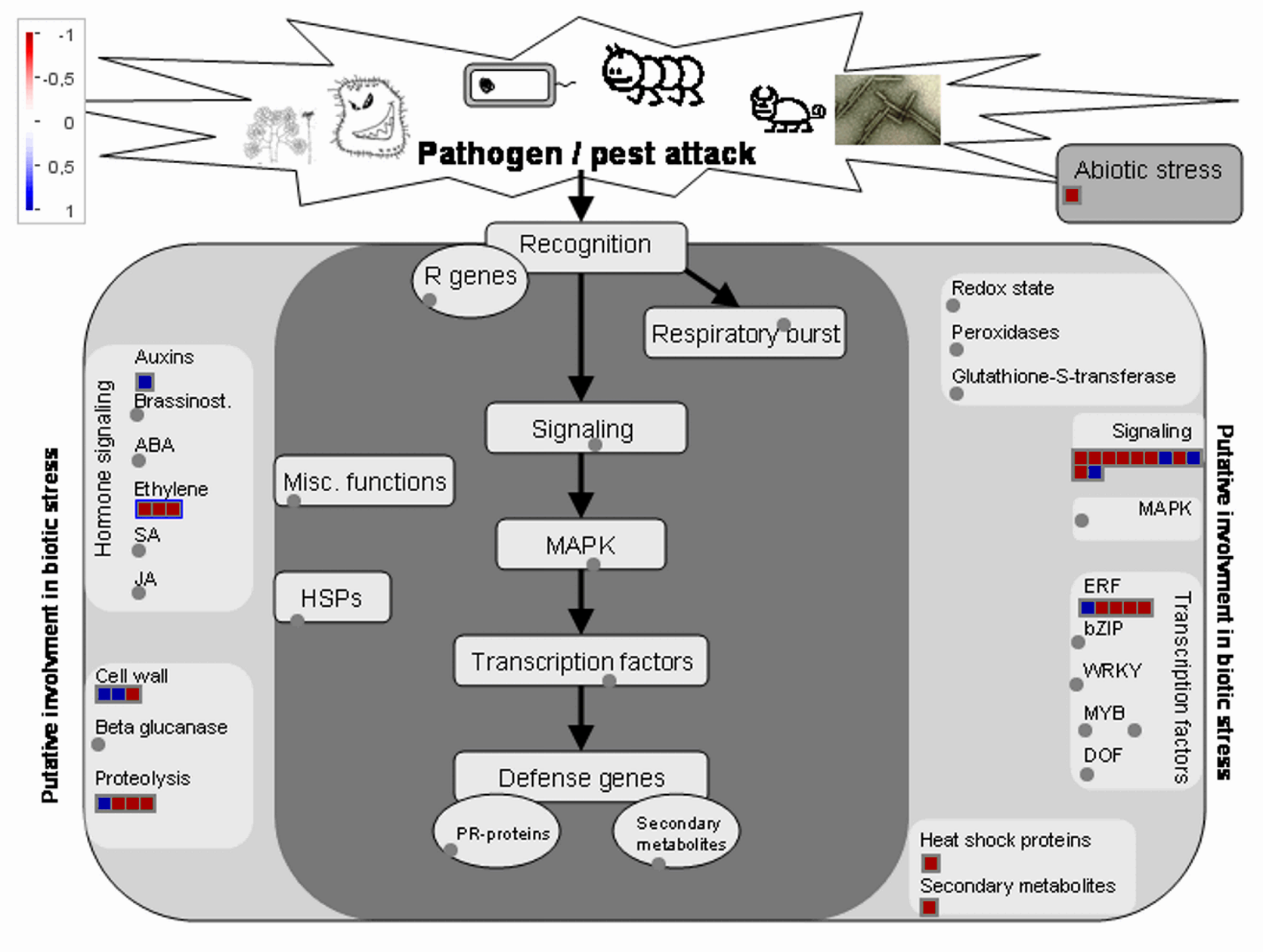

Supplement: S7 Fig — Transcripts of Hevea brasiliensis associated to biotic stress from Ricinus communis (affimetrix arrays). From 86 differential expressed genes 74 were mapped (some of the data points may be mapped multiple times to different bins) and visible in this pathway: 30 (TIF) [file pone.0134837.s007.tif]
